# Response of cell-wall composition and RNA-seq transcriptome to methyl-jasmonate in *Brachypodium distachyon* callus

**DOI:** 10.1007/s00425-018-2968-9

**Published:** 2018-08-09

**Authors:** Lucy S. Hyde, Till K. Pellny, Jackie Freeman, Louise V. Michaelson, Rachael Simister, Simon J. McQueen-Mason, Rowan A. C. Mitchell

**Affiliations:** 1 Plant Sciences Department, Rothamsted Research, Harpenden, Herts AL5 2JQ UK; 2 Centre for Novel Agricultural Products (CNAP), Department of Biology, University of York, York, YO10 5DD UK

**Keywords:** Arabinoxylan, Coumaroylation, Hemicellulose, Hydroxycinnamic acids, Jasmonic acid, Lignin

## Abstract

Methyl-jasmonate induces large increases in p-coumarate linked to arabinoxylan in Brachypodium and in abundance of GT61 and BAHD family transcripts consistent with a role in synthesis of this linkage.

Jasmonic acid (JA) signalling is required for many stress responses in plants, inducing large changes in the transcriptome, including up-regulation of transcripts associated with lignification. However, less is known about the response to JA of grass cell walls and the monocot-specific features of arabinoxylan (AX) synthesis and acylation by ferulic acid (FA) and *para*-coumaric acid (*p*CA). Here, we show that methyl-jasmonate (MeJA) induces moderate increases in FA monomer, > 50% increases in FA dimers, and five–sixfold increases in *p*CA ester-linked to cell walls in Brachypodium callus. Direct measurement of arabinose acylated by *p*CA (Ara*f*-*p*CA) indicated that most or all the increase in cell-wall *p*CA was due to *p*CA ester-linked to AX. Analysis of the RNA-seq transcriptome of the callus response showed that these cell-wall changes were accompanied by up-regulation of members of the GT61 and BAHD gene families implicated in AX decoration and acylation; two BAHD paralogues were among the most up-regulated cell-wall genes (seven and fivefold) after 24 h exposure to MeJA. Similar responses to JA of orthologous BAHD and GT61 transcripts are present in the RiceXPro public expression data set for rice seedlings, showing that they are not specific to Brachypodium or to callus. The large response of AX-pCA to MeJA may, therefore, indicate an important role for this linkage in response of primary cell walls of grasses to JA signalling.

**Electronic supplementary material:**

The online version of this article (10.1007/s00425-018-2968-9) contains supplementary material, which is available to authorized users.

## Introduction

Jasmonic acid (JA) is a lipid-derived phytohormone and signalling molecule involved in plant development and in response to biotic and abiotic stresses. JA orchestrates a complex signalling cascade, involving cross-talk with other hormones such as ethylene, abscisic acid, and salicylic acid, which activates transcription factors controlling defence genes, such as protease inhibitors, terpenoids, phytoalexins, flavonoid, and sesquiterpenoid biosynthesis enzymes and antifungal proteins (Creelman and Mullet 1995; Avanci et al. 2010; Wasternack and Hause 2013).

The effects of JA signalling are often studied by the exogenous application of methyl–JA (MeJA), which is cleaved by MeJA esterase to JA *in planta* (Wu et al. 2008). Activation of JA-responsive genes requires conversion of JA to its bioactive isoleucine conjugate (JA-Ile). JA-Ile binds to the Skp1–Cullin–F-box (SCF)^COI1^ E3 ubiquitin ligase complex triggering the degradation of JAZ transcriptional repressor proteins, which normally repress the activity of the MYC2 transcription factor in the nucleus, resulting in the expression of JA-responsive genes. This system has been demonstrated in Arabidopsis (Thines et al. 2007; Chini et al. 2007) and has, to some extent, been shown to be conserved in rice (Lee et al. 2013). Studies have reported the effects of exogenously applied MeJA on global transcription, in both dicots and monocots: Pauwels et al. (2008) report that 6-h MeJA induced differential expression of 495 genes in cell suspension cultures of Arabidopsis; Salzman et al. (2005) report that MeJA induced and down-regulated expression (> 1.5-fold) of 2980 and 1842 genes, respectively, in *Sorghum*; and transcriptome response to JA of rice seedlings was profiled as part of construction of the public expression database RiceXPro (Sato et al. 2013). Transcripts for enzymes in the phenylpropanoid pathway (e.g., 4CL, COMT, CCR, CAD, and CCoAOMT) leading to the synthesis of monolignols were significantly up-regulated by JA in all these studies. The mechanism of up-regulation of lignin biosynthetic genes in maize is now known to be analogous to that described for Arabidopsis above; the maize genes contain cis elements that bind to repressors for which degradation is triggered by JA signalling (Vélez-Bermúdez et al. 2015). In Arabidopsis cell suspension cultures, the increase in lignin biosynthetic transcripts was accompanied by a progressive increase in cellular monolignol content after MeJA treatment (Pauwels et al. 2008). Lignin polymerisation from monolignols is dependent on cell-wall class III peroxidases, which generate reactive oxygen species (ROS) from hydrogen peroxide. Peroxidase expression and activity is also known to be drastically increased by JA (Almagro et al. 2009). These cell-wall-related changes in response to JA do not necessarily result in detectable increases in total lignin, but are rather associated with a cessation of growth (Napoleao et al. 2017), including decreased cell expansion which may be due to increased cross-linking of primary cell walls.

In commelinid monocotyledons, including the major grass cereal crops wheat, rice, and maize, cell walls contain xylan with abundant arabinofuranose decorations (arabinoxylan; AX). which can be acylated on the O-5 position by hydroxycinnamic acids (HCAs) ferulic acid (FA) or *para*-coumaric acid (*p*CA). AX-FA oxidatively couples to form dimers and/or cross-links to lignin in the presence of ROS (Ralph et al. 1995), whereas the role of AX-*p*CA is less clear as it participates much less in cross-links (Ralph 2010). Despite the importance of FA and *p*CA in the grass cell wall, the mechanism by which these phenolic acids become ester-linked to AX remains unclear. We predicted that a clade of genes within the BAHD superfamily of acyl-coA transferases would contain the genes responsible for feruloylation of AX (Mitchell et al. 2007). Subsequently, other groups have shown that some of these genes actually add *p*CA [PMT; (Withers et al. 2012; Petrik et al. 2014; Sibout et al. 2016)] or FA [FMT; (Karlen et al. 2016)] to monolignols. However, there is strong evidence that one of the genes in this clade, *OsAT10*, is responsible for acylation of AX with *p*CA in rice as specific up-regulation of this gene increased Ara*f*-*p*CA fivefold (Bartley et al. 2013). RNAi suppression of other genes in this clade resulted in decreased cell-wall FA (nearly all of which is likely to be AX-FA) (Piston et al. 2010; Buanafina et al. 2016) with the strongest effect resulting from suppression of a gene we call *SvBAHD01* in *Setaria viridis* (de Souza et al. 2018). Genes within glycosyl transferase family 61 (GT61) are responsible for the addition of three-linked Araf on AX (Anders et al. 2012) and a knock-out mutant for a GT61 gene *xax1* had severely decreased wall-bound FA and *p*CA (Chiniquy et al. 2012). Since BAHD proteins are localised in the cytosol, where the donor molecule for arabinosylation of AX, UDP-Araf, is synthesised (Konishi et al. 2007; Rautengarten et al. 2011), one model is that BAHD proteins are responsible for the addition of HCA ester-linked to this donor molecule before it is transported into the Golgi, where XAX1 protein mediates the addition of Araf-HCA onto AX (Buanafina 2009; Molinari et al. 2013). This model is not universally accepted; Chiniquy et al. (2012) interpreted their data differently and the existence of the putative UDP-Araf-HCA intermediate has not been reported. Nevertheless, there is extensive circumstantial evidence for the involvement of these BAHD and GT61 genes in the addition of HCA to AX (Mitchell et al. 2007; Bartley et al. 2013; Buanafina et al. 2016; de Souza et al. 2018).

Evidence from the public expression database RiceXPro shows that several genes in the BAHD and GT61 candidate clades have dramatically increased expression in response to JA in rice seedlings grown hydroponically (Sato et al. 2013). We hypothesised that this increased expression would result in increased abundance of AX-FA and/or AX-*p*CA in cell walls. Recently, it has been shown that treatment with MeJA does induce small, but significantly increases in cell-wall FA and *p*CA in leaves of *Brachypodium distachyon* (Brachypodium) (Napoleao et al. 2017) and we have similar findings (L. S. Hyde, unpublished). However, leaves contain a complex mix of primary and secondary cell walls that contain both Ara*f*-*p*CA and lignin-*p*CA. Therefore, we chose to examine the effects of MeJA on primary cell-wall composition in Brachypodium callus as a more tractable system.

## Materials and methods

### Callus growth and harvesting

*Brachypodium distachyon* (L.) P. Beauv. callus was generated and sub-cultured as previously described (Vogel and Hill 2008). Tissue was harvested directly into liquid nitrogen and ground to a fine powder using a Spex SamplePrep Freezer/Mill, or by hand using a pestle and mortar. Tissue was stored at − 80 °C for RNA extraction or freeze-dried for cell-wall composition analyses.

### Methyl-jasmonate treatment

Experiment 1: sub-cultured callus was transferred onto plates of callus initiation media [CIM; 4.43-g/l LS salts, 30-g/l sucrose, 0.6-mg/l CuSO_4_, 2.5-mg/ml 2,4-D, 0.2% (w/v) Phytagel™, pH 5.8] containing 1-, 5-, 10-, 50-, and 100-µM MeJA (in ethanol) and a mock control (ethanol). Nine calli per plate (plate = 1 biological replicate) with three biological replicates per treatment.

Experiment 2: as experiment 1, except 16 calli per plate (plate = 1 biological replicate) with four biological replicates per treatment.

Experiment 3: Brachypodium calli were transferred onto plates of CIM containing 50-µM MeJA (in ethanol), or ethanol as a mock control. Samples were taken at 24 and 48 h, and 4 and 8 days. Three plates of 36 calli per plate were pooled per treatment, per timepoint and four biological replicates were analysed. Additional replicates were generated to measure the proportion of ester-linked HCA in the pellet and supernatant fractions after mild acid hydrolysis and were harvested after 7 days of MeJA treatment.

Experiment 4: Brachypodium calli were treated with 50-µM MeJA as in Expt. 3, except that the same callus was divided and transferred to control and MeJA plates in a paired design that better corrects for variation between the original calli.

### Biochemical analyses

Experiment 1: phenolic acid content was quantified as previously described and expressed on a dry weight basis (Pellny et al. 2012).

Experiment 2: phenolic acid content was quantified as above. For all other analyses, destarched alcohol-insoluble residue (AIR) was extracted from three biological replicates per treatment. Tissue (20–50 mg) was washed successively with phenol, chloroform:methanol (2:1, v/v), and ethanol. The resulting pellet was air-dried for 2 h. Starch was removed using a method slightly modified from (Harholt et al. 2006). The AIR pellet was suspended in 10-mM potassium phosphate buffer, pH 6.5, 1-mM CaCl_2_, 0.05% (w/v) NaN_3_, preheated to 95 °C. After 30 s, 1 unit/ml α-amylase (*Bacillus lichenformis*, Sigma-Aldrich) was added and samples were incubated at 85 °C for 15 min. The destarched AIR was collected by centrifugation (> 10,000*g*, 20 min), washed thrice with ethanol and dried at 60 °C under vacuum. Matrix monosaccharides were analysed as previously described (Jones et al. 2003) and the remaining pellet was using to quantify cellulose using a method modified from Viles and Silverman (1949): the pellet was washed with water once and with acetone thrice. Cellulose was hydrolysed in 72% (w/v) aqueous sulphuric acid at room temperature for 4 h, and in 3.2% (w/v) aqueous sulfuric acid at 120 °C, for 4 h. After centrifugation, 40 µl of the supernatant was diluted with 360-µl water and added to 800-µl sulfuric acid containing 2-mg/ml anthrone reagent. Samples were heated at 80 °C for 30 min. Absorbance was read at 620 nm and compared to the absorbance of glucose standards.

Experiment 3: AIR was prepared as described by Goubet et al. (2009), except tissue (100 mg) was prepared by hand grinding in liquid nitrogen and was freeze-dried. AIR was destarched following a method slightly modified from Englyst et al. (1994). AIR (10 ± 0.20 mg) was suspended in 0.1-M sodium acetate buffer, pH 5.2, with 1.25% (v/v) α-amylase (*Bacillus lichenformis*, Sigma-Aldrich) and incubated at 85 °C, for 1 h, with shaking. Pullulanase (5 µl, *Bacillus acidopullulyticus*, Sigma-Aldrich) was added and incubated at 50 °C for 30 min, with shaking. Polysaccharides were precipitated in 1.3-ml cold ethanol for 1 h on ice, pelleted by centrifugation (10,000*g*, 4 °C, 10 min), and washed thrice in 70% (v/v) aqueous ethanol. The pellet was dried at 40 °C under vacuum. Destarched AIR was analysed for phenolic acid composition, matrix monosaccharides, and cellulose as above, and acetyl bromide lignin as previously described by Foster et al. (2010) and results expressed per unit destarched AIR. Quantification of HCA content of the pellet and supernatant fractions after mild acidolysis was by incubation of dried AIR t in 0.6-ml 0.05-M trifluoroacetic acid (TFA) at 100 °C for 4 h, with shaking. Samples were centrifuged (10,000*g*, 10 min) and 500-µl supernatant and the pellet, after three washes with water, were dried under vacuum at 40 °C. Internal standard and 2-M NaOH were added and phenolic acids were extracted and analysed as above.

Experiment 4: AIR was prepared by washing Brachypodium callus in 80% (v/v) aqueous ethanol as described by Pellny et al. (2012). The pellet was subsequently washed in CHCl_3_:MeOH (3:2) and dried for 16 h at 60 °C with tube lids open. Total cell-wall-bound phenolic acid measurements were as described above. Quantification of Ara-HCA and HCA released by mild acidolysis was by incubation of dried AIR in 1.2-ml 0.05-M trifluoroacetic acid (TFA) at 100 °C for 2 h, with shaking. Samples were centrifuged (14,000*g*, 10 min) and two aliquots of 500-µl supernatant were freeze-dried. Internal standard and 2-M NaOH was added to one aliquot of supernatant, and phenolic acids were extracted and analysed as above. The other aliquot of supernatant was analysed for Ara-HCA content using the LC–MS method described in (de Souza et al. 2018) except that here, quantification was achieved from the ion count of the multiple-reaction monitoring (MRM) rather than the associated UV absorbance peak (as there were overlapping UV absorbance peaks in these samples). We previously isolated fractions of Ara-HCA and quantified HCA in these (de Souza et al. 2018); from ion counts of Ara-HCA MRMs for these, we were able to estimate conversion factors from ion counts to HCA amounts under our conditions.

### RNA sequencing

RNA was extracted from 32 samples generated in Expt. 3 (2 treatments × 4 timepoints × 4 reps) as previously described by Chang et al. (1993). RNA sequencing was performed on an Ion Proton™ System. Libraries were made using the Ion Total RNA-Seq Kit v2, and templates were prepared using the Ion PI™ Template OT2 200 Kit V2 and were sequenced using the Ion PI™ Sequencing 200 Kit v2 with an Ion PI™ Chip Kit v2. All sequencing equipment and reagents were from Thermo Fisher Scientific and used following the manufacturer’s instructions. Sequencing reads were analysed on the Galaxy platform (Giardine et al. 2005). Reads were mapped to the *Brachypodium distachyon v3.1* reference transcriptome from Phytozome 11.0 (Goodstein et al. 2012) with one representative splice variant per gene. Comparison with an earlier reference Genebuild 2010-02-Brachy 1.2 showed that the transcript for candidate gene *BdBAHD04*, *BRADI2G33980.1* was replaced by a transcript from the opposite strand, *Bradi2g33977.1* in v3. However, the strand-specific Ion Torrent reads all mapped to the strand in the v1.2 gene model, hence, the *Bradi2g33977.1* sequence in the v3.1 reference was manually replaced with *BRADI2G33980.1*, and this was used for all results reported here. Reads less than 30 bp were removed using the Trimmomatic tool, and the remainder mapped to the reference transcriptome with BWA-MEM, and percentage mapped reads were obtained using Flagstat. Mapped reads were quantified using eXpress, and tables of effective counts and FPKM (fragments per kilobase of transcript per million mapped reads) were created using Merge eXpress. For global analysis, ANOVA was applied on effective counts, performed in RStudio using the EdgeR package, taking account of the four biological replicates per sample. This analysis tested for the main effects and interaction between the two factors treatment and time, at the *P* = < 0.05 significance level corrected for multiple testing using Benjamini–Hochberg false-discovery rate, after filtering for genes with counts per million > 1 in three samples or more. For cell-wall genes analysis, a set of 492 cell-wall genes listed in Table S4 were identified from their gene families using characteristic domains identified in Ensembl Plants or from genes listed in Plant Metabolic Network database (PMN) for the phenylpropanoid pathway; Arabidopsis and rice orthologues from Ensembl Plants were used to check gene family assignment using TAIR and CAZy. ANOVA was performed as above on only these genes to determine differentially expressed cell-wall genes.

## Results

### Effect of MeJA concentration

**Fig. 1:**
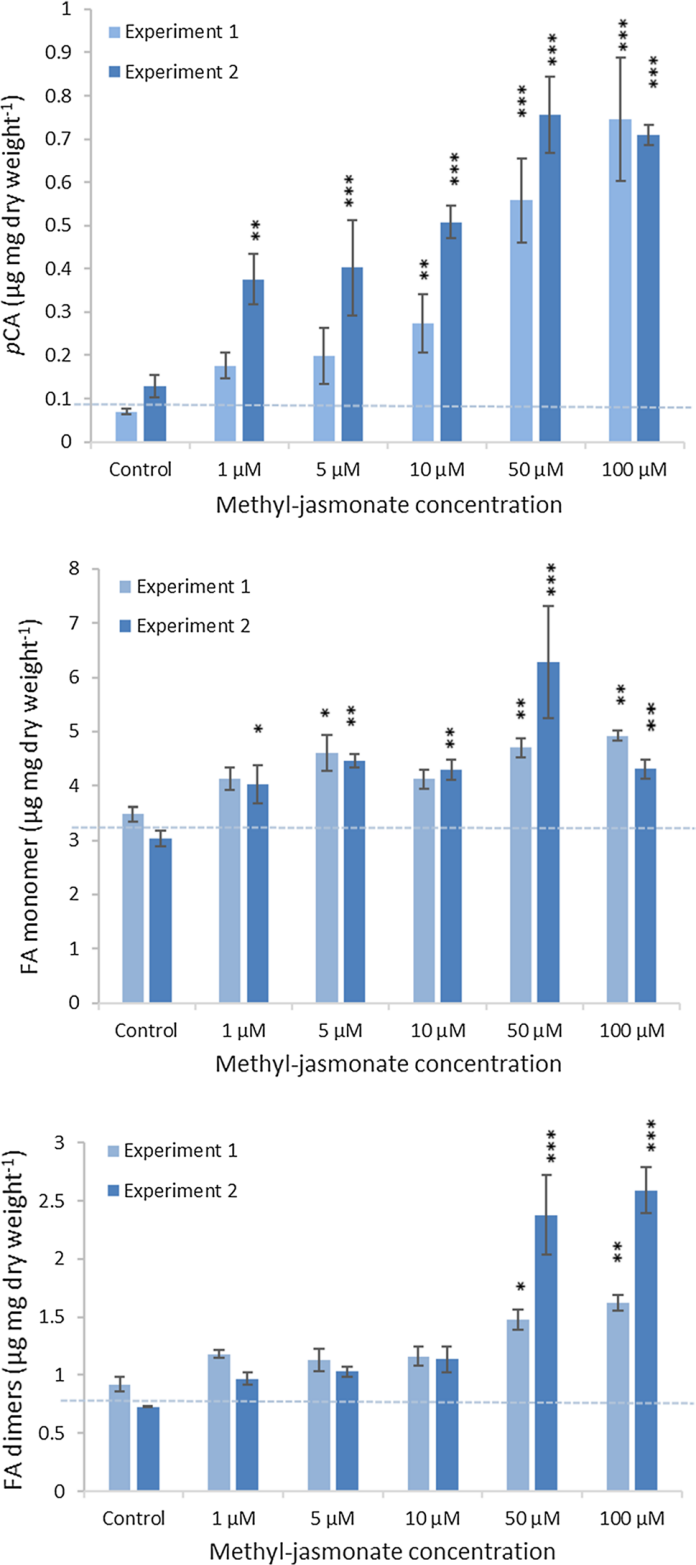
Effect of 17-day treatment with increasing concentrations of MeJA on bound *p*CA and FA monomer and dimers ± SE, in two experiments (*n *= 3 Expt. 1, *n *= 4 Expt. 2). FA dimers are the sum of diF8-8′, diF8-5′, diF8-5′ benzofuran, diF5-5′ and diF8-O-4′. Significance of differences from control level are indicated, where these are greater than maximum LSD from ANOVA of all data with *(*P *< 0.05), **(*P *< 0.01), ***(*P *< 0.001)

**Fig. 2:**
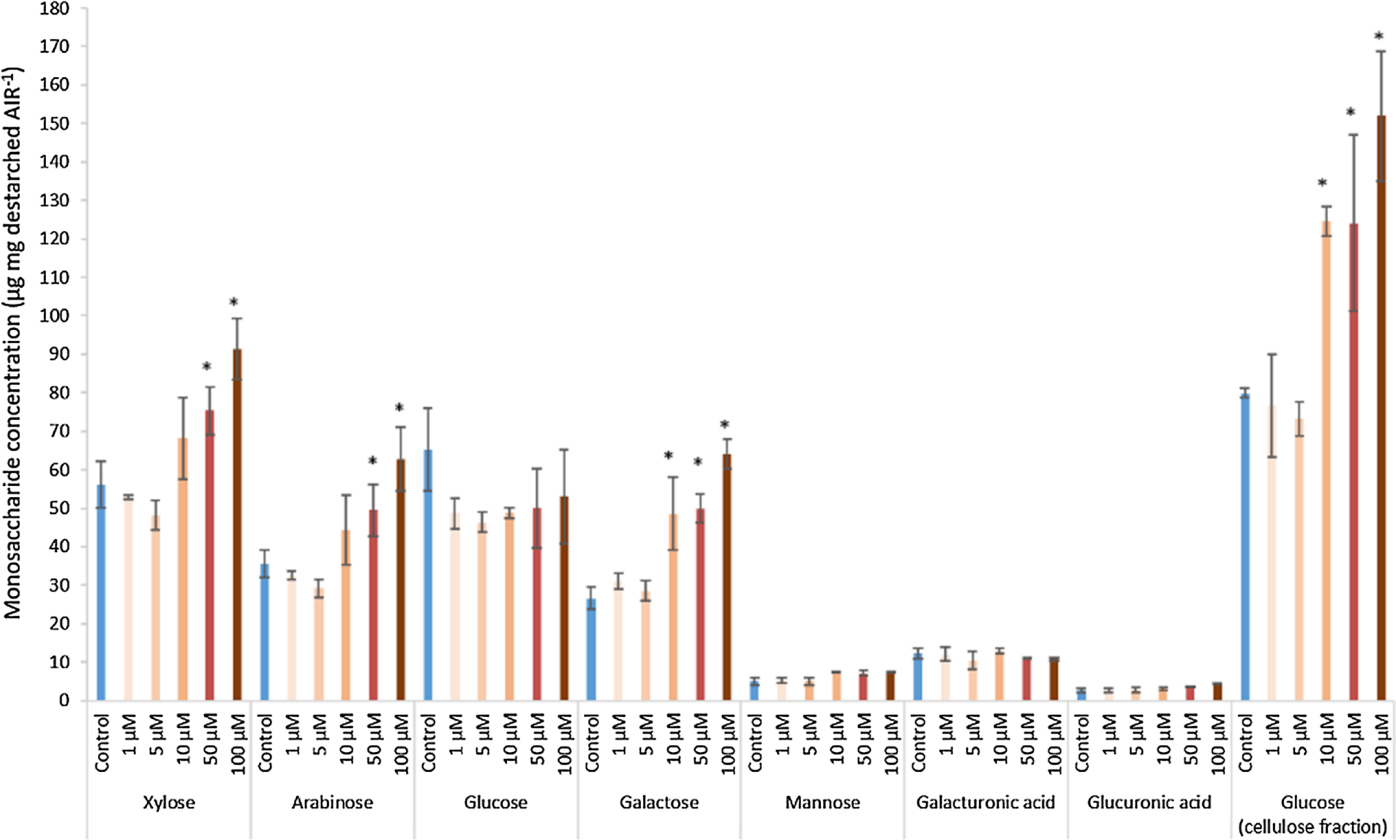
Monosaccharide concentrations ± SE in the hemicellulose fraction (xylose, arabinose, glucose, galactose, mannose, galacturonic acid, and glucuronic acid) and glucose in the cellulose fraction of destarched alcohol-insoluble residue (AIR) in Brachypodium callus after 17-day treatment with varying concentrations of methyl-jasmonate (1, 5, 10, 50, and 100 µM, Expt. 2, *n *= 4). * indicates significant difference from control level (*P *< 0.05)

We investigated the effect of increasing concentrations of MeJA (1–100 µM) on Brachypodium callus cell walls after 17-day treatment; the highest MeJA concentrations noticeably slowed callus growth (Fig. S1). Bound hydroxycinnamic acid content was increased by MeJA treatment in two experiments (Fig. 1); significant increases were observed for *p*CA, and FA monomer in Expt. 2 even at 1-µM MeJA (*P* < 0.05 for all) with maximal increases for *p*CA, and FA monomer and dimers observed at 50 or 100 µM. Bound *p*CA showed the largest increases relative to control, of five–ninefold at 100-µM MeJA (*P* < 0.001), whereas the increase in FA monomer was 42% (*P* < 0.01) and increase in FA dimers was 76–350% (*P* < 0.01). We analysed cell-wall sugars from Expt. 2 (Fig. 2). At concentrations of MeJA of 10 µM and above, cellulosic glucose and galactose were increased compared to control samples. At 50- and 100-µM MeJA, arabinose and xylose were significantly (*P *< 0.05, LSD) greater than control as a proportion of alcohol-insoluble residue (AIR). No statistically significant change was observed in hemicellulosic glucose, mannose, galacturonic acid, or glucuronic acid.

### Time course of MeJA effects: cell-wall composition

**Fig. 3:**
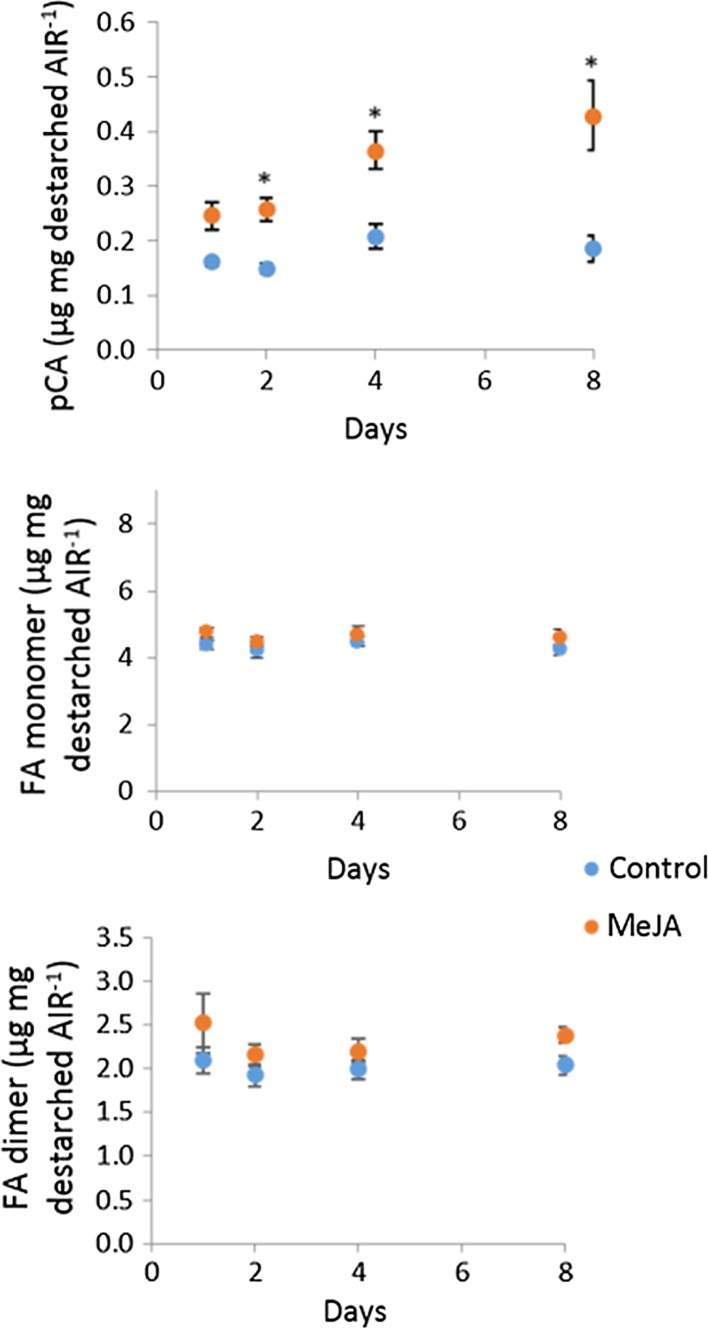
Effect of 1-, 2-, 4-, and 8-day treatment with 50-µM MeJA on bound pCA, FA monomer and dimers ± SE in Brachypodium callus cell walls in Expt. 3 (*n *= 4). FA dimers are the sum of diF8-8, diF8-5, diF8-5 benzofuran, diF5-5, and diF8-O-4. * indicates difference between control and MeJA is greater than LSD from ANOVA (*P *< 0.05)

**Fig. 4:**
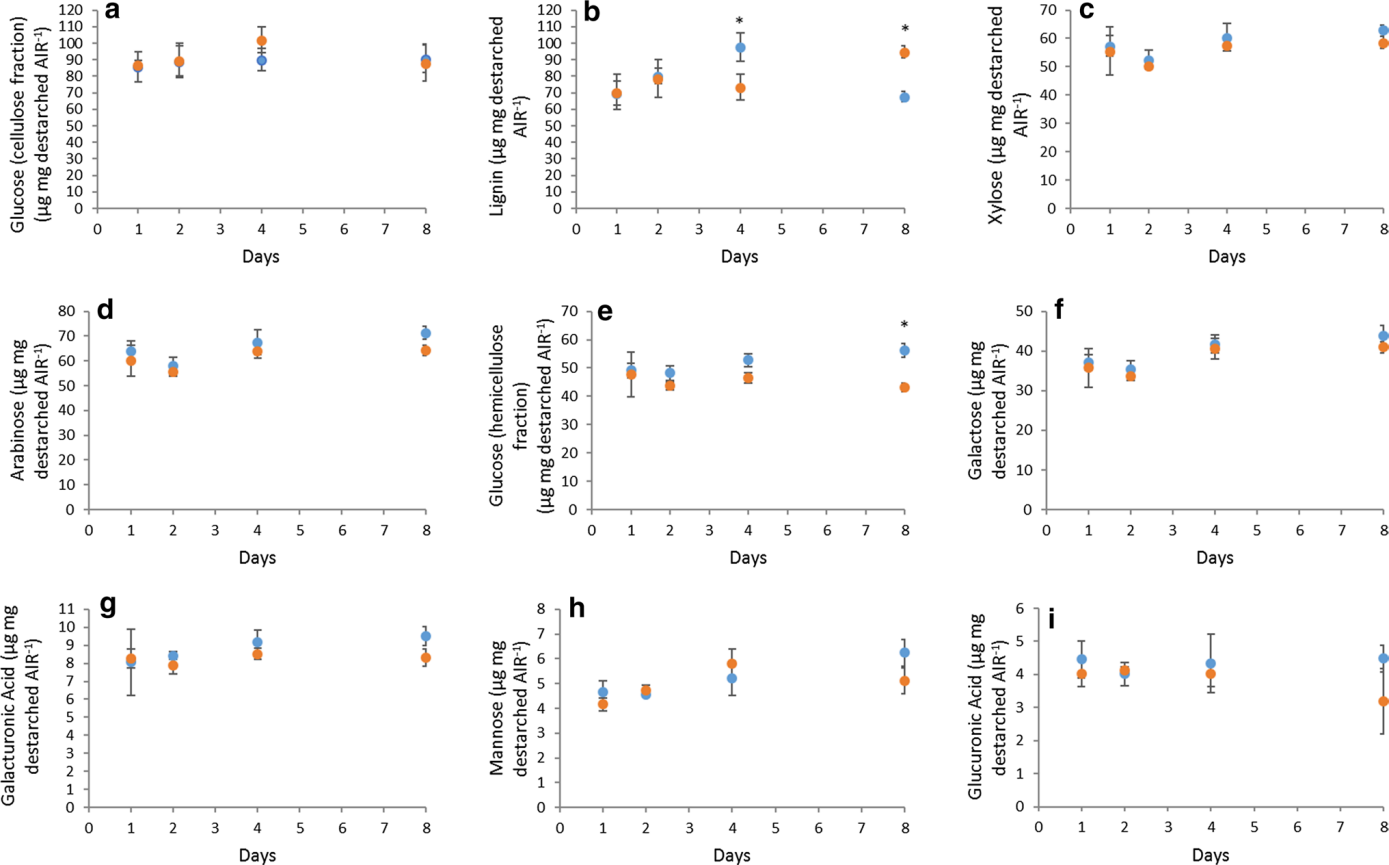
Effect of 50-µM methyl-jasmonate (MeJA) on cell-wall polysaccharide (**a**, **c**–**i**) and lignin (**b**) composition in *Brachypodium distachyon* callus destarched AIR (alcohol-insoluble residue) after 1-, 2-, 4-, and 8-day treatment in Expt. 3 (*n *= 4). Blue and orange markers represent control and MeJA samples, respectively. Error bars show ± SE. * indicates difference between control and MeJA is greater than LSD from ANOVA (*P *< 0.05)

We investigated the effects of treatment with 50-µM MeJA on Brachypodium callus sampled at 24 and 48 h, and 4 and 8 days in two experiments. In the first of these (Expt. 3), we compared cell-wall composition and the RNA-seq transcriptome; in Expt. 4, we examined effects on cell-wall hydroxycinnamate in more detail. For these experiments, we express cell-wall composition as a proportion of cell-wall fraction (AIR or destarched AIR); in fact, the proportion of callus dry weight (DW) present as AIR (40–42%) and as destarched AIR (19–22%) was not altered by MeJA treatment (Table S1), so the relative effects of MeJA expressed per unit DW (Fig. 1) or per unit AIR (Figs. 2, 3, 4) are comparable.

Bound *p*CA accumulated rapidly in Brachypodium callus when treated with 50-µM MeJA (Fig. 3); *p*CA was 50% greater than the control samples after 24 h (*P* < 0.05, LSD), and continued to accumulate, increasing significantly to twofold greater than the control by day 8 of treatment (*P* < 0.05, LSD). There was a significant main effect of MeJA over time on wall-bound FA monomer (*P *= 0.03, *F* test), although this effect was small; MeJA-treated samples remained 5–9% greater than the control over 1–8-day treatment. We found significantly (*P *= 0.018, *F* test) greater total wall-bound FA dimers in MeJA-treated samples (Fig. 3c).

Individual FA dimers (diFAs) showed similar relative responses to any of the MeJA treatments in the four experiments (Table S2). This contrasts with markedly differing responses of different diFA dimers to suppression of *SvBAHD01* gene *Setaria viridis* (de Souza et al. 2018).

We also determined monosaccharide composition of the cell-wall fraction (destarched AIR) in Expt. 3 (Fig. 4). The only consistent, significant effect of 50-µM MeJA treatment was a decrease in hemicellulosic glucose, presumably (1,3;1,4)-β-glucan (*P *= 0.016, *F* test). There was no significant effect on hemicellulose associated xylose, arabinose, galactose, galacturonic acid, mannose or glucuronic acid, or in cellulosic glucose.

**Fig. 5:**
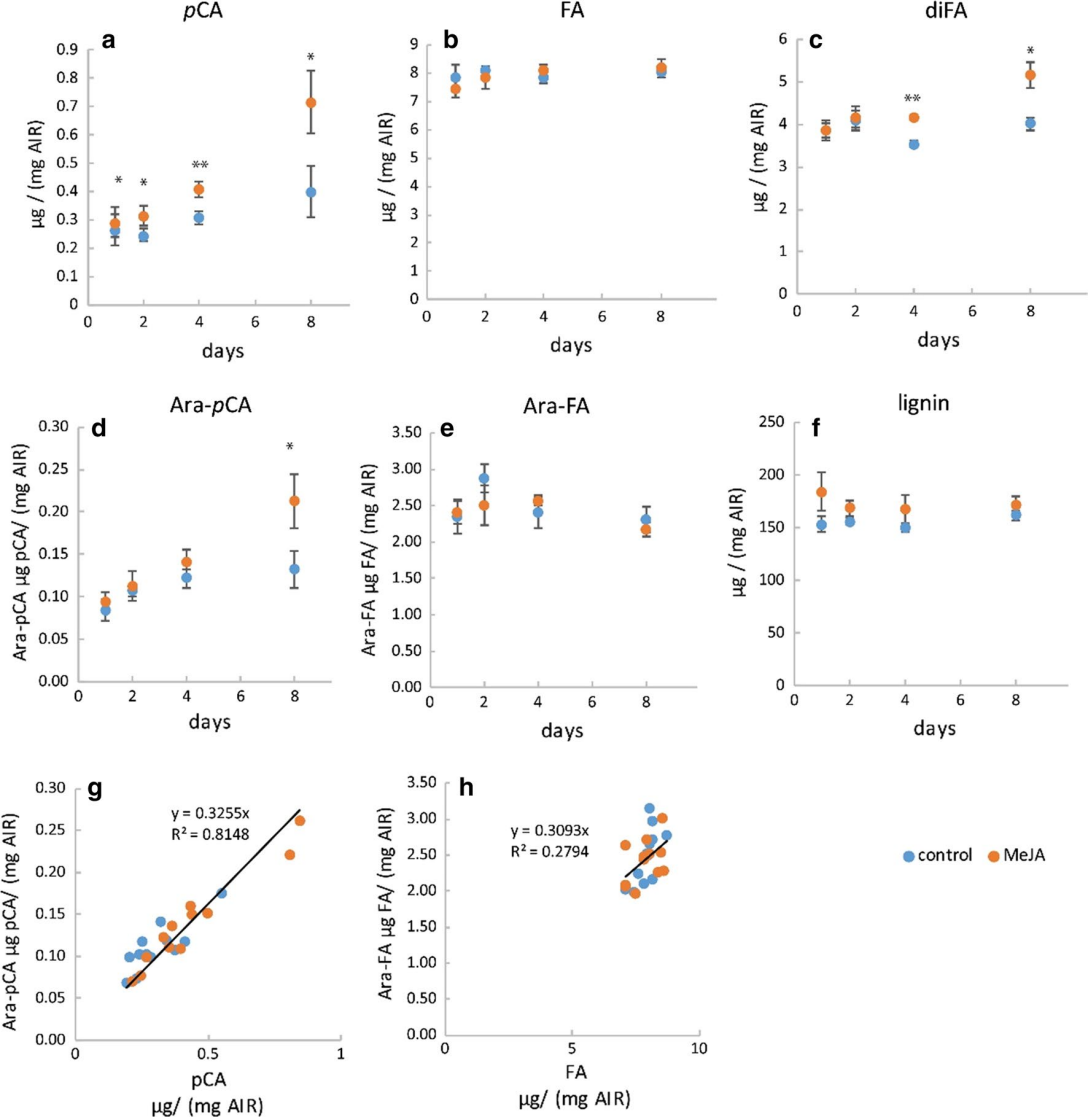
Effect of 50-µM methyl-jasmonate (MeJA) on cell-wall HCA (**a–c**) and lignin (**f**) content in *Brachypodium distachyon* callus AIR (alcohol-insoluble residue) after 1-, 2-, 4-, and 8-day treatment (Expt. 4). HCA content released by saponification (**a–c**) including total FA dimer content (**c**). FA dimers are the sum of diF8-8 aryltetralin, diF8-8′, diF8-5′, diF8-5′ benzofuran, diF5-5′, and diF8-O-4′ (individual diFA data in Table S2). Determination by LC–MS of Ara-HCA conjugates released by mild acidolysis (**d**, **e**). Relationship between *p*CA and Ara*f*-*p*CA (**g**) and FA and Ara*f*-FA (**h**) content. Points with error bars (**a–f**) show mean ± SE, *n *= 3; * and ** indicate significant difference between control and MeJA from paired *t* test at *P *< 0.05, 0.01, respectively. Points in **g**, **h** show individual sample values

In Expt. 3, we found that lignin content of destarched AIR did not show a consistent trend with MeJA treatment but had significantly (*P* < 0.05) greater lignin at 8 days than controls (Fig. 4b). We also analysed lignin in Expt. 4, and found that whilst no individual timepoint was significantly different, overall, there was a significant increase (*P* < 0.05) in MeJA-treated relative to controls (Fig. 5f).

The *p*CA ester-linked to cell walls in grasses is made up of both *p*CA ester-linked to lignin and that ester-linked to AX, with lignin-*p*CA being the more abundant form in most tissues (Petrik et al. 2014; de Souza et al. 2018). To find which form is increased by MeJA, we used mild acidolysis to release sugar-linked HCA from AIR. Application of this treatment to plant tissues results in most lignin-*p*CA being left in the pellet; we found only a small proportion of ester-linked *p*CA in the pellet fraction from these callus samples (Table S3) suggesting that most of it is present as AX-*p*CA. However, there was a possibility that lignin-*p*CA in callus differs from plant tissues and is solubilised by mild acidolysis. We addressed this in Expt. 4 using our method for analysing Ara-HCA (de Souza et al. 2018), where we found that Ara*f*-*p*CA was increased by MeJA treatment (Fig. 5d) showing that AX-*p*CA increases in response to MeJA. We found similar relative increases in Ara*f*-*p*CA (Fig. 5d) to that for total ester-linked *p*CA (Fig. 5a). There are losses inherent in the mild acidolysis treatment, and correlating Ara*f*-*p*CA to total *p*CA across all samples, we recovered about 33% as Ara*f*-*p*CA (Fig. 5g). We found a similar proportion of total ester-linked FA monomer present as Ara*f*-FA (Fig. 5h) and a similar proportion of ester-linked FA in the pellet fraction after mild acidolysis (Table S3). Since all FA released by saponification are expected to come from AX-FA, this suggests that all, or nearly all, the *p*CA released by saponification in the callus samples come from AX-*p*CA.

### Time course of MeJA effects: RNA-seq transcriptome

The transcriptome of samples from Expt. 3 (callus treated with 50 µM of MeJA for 24 and 48 h, and 4 and 8 days) was analysed by RNA-seq. We obtained an average of 6.8 million reads per sample of which 93% mapped to the reference. A multidimensional scaling factor (MDS) plot showed that MeJA treatment had a large effect on the variation in the transcriptome between samples, whereas time resolved the variation to a much lesser extent; replicates all grouped according to MeJA treatment on the MDS plot (Fig. S2). Differentially expressed genes (DEGs) were defined as those with significant effects of treatment, time, or treatment:time interaction at *P *< 0.05 with Benjamini–Hochberg false-discovery rate correction. Out of a total of 5695 DEGs (Table S4), 4508 were induced by the MeJA treatment factor, 1270 DEGs for the time factor, and only 170 genes that showed an interaction effect between treatment and time, with some overlap between these gene sets (Fig. S3). Within the treatment DEGs, 2034 genes were up-regulated and 1985 genes were down-regulated at every timepoint.

We examined transcript abundance from a set of 483 genes identified as putatively encoding enzymes for synthesising cell-wall constituents or cell-wall proteins (Table S5). Forty of these cell-wall-synthesis genes were significantly up-regulated and twenty-two down-regulated in response to 50-µM MeJA; only one of these was also significantly affected by time and none showed a significant time:treatment interaction, so all cell-wall genes were stably affected by MeJA during the 8-day time course. The high level of replication (*n *= 4) and relative simplicity of effect give us good statistical power, so any effect of MeJA > = 1.4-fold was highly significant. The up-regulated set of 40 DEGs included genes from glycosyl transferase families (GT) GT2, GT4R, GT8, GT31, GT61, GT64, GT65, and GT77, genes from the BAHD clade and phenylpropanoid pathway genes (Table 1). Apart from one cinnamoyl-coA reductase (CCR) gene, the most up-regulated transcripts at the 24-h timepoint were two BAHD paralogs (*Bradi2g04980*, *Bradi2g04990*) within the Clade that we previously identified as having a possible role in AX feruloylation (Mitchell et al. 2007). Other highly up-regulated transcripts (> twofold at 24 h) include another member of this BAHD Clade (*Bradi2g33980*), putatively encoding phenylpropanoid pathway enzymes (4CL, HCT, CCR), and members of the GT61, GT77, and GT31 families. More moderately up-regulated (> = 1.4-fold, < twofold at 24 h) genes include other BAHD and GT61 candidates and genes implicated in xylan backbone synthesis and cellulose synthesis (Table 1).

**Table 1:** Transcript abundance in FPKM (average of 4 reps) of up-regulated cell-wall genes after 1-, 2-, 4-, or 8-day treatment with 50-µM MeJA (JA) compared to a mock control (MC)

Transcript	Candidate name/putative function/family [source]	24 h			48 h			4 days			8 days			Overall JA *P* value
		MC	JA	JA/MC (%)	MC	JA	JA/MC (%)	MC	JA	JA/MC (%)	MC	JA	JA/MC	
Bradi3g19670.1	Cinnamoyl-CoA reductase [ortho ATCCR2]	0.2	16.0	6978	0.0	7.9	17,649	0.0	5.4	–	0.0	3.0	7060	3.5E−13
Bradi2g04980.1	BdBAHD02p2	0.3	2.0	774	0.2	1.4	557	0.4	1.2	300	0.5	1.7	344	2.3E−07
Bradi2g04990.1	BdBAHD02p1	1.1	6.1	536	1.0	5.8	591	1.4	5.8	422	2.1	5.8	275	2.5E−11
Bradi3g37300.1	4-coumarate-CoA ligase (4CL) [ortho Os4CL5]	1.8	8.6	483	0.9	5.8	615	1.1	6.6	594	1.7	5.7	332	1.0E−08
Bradi2g01380.1	BdGT61_21	0.3	1.2	458	0.2	1.0	565	0.5	1.3	251	0.2	1.1	459	2.0E−04
Bradi1g15590.1	GT31 family [ortho AtB3GALT1]	0.4	1.8	423	0.7	1.7	233	1.2	2.0	164	1.1	1.9	164	7.8E−05
Bradi2g58987.1	GT family 77 [CAZy]	0.9	3.6	386	0.9	2.3	268	0.7	1.9	268	0.5	1.6	337	4.1E−05
Bradi1g76170.1	4-coumarate-CoA ligase (4CL) [PMN]	13.7	26.9	197	16.4	40.1	244	19.1	35.1	184	21.5	41.1	191	1.8E−06
Bradi2g23740.1	hydroxycinnamoyl-coA shikimate transf. [PMN]	0.8	1.6	196	0.8	2.2	268	1.4	2.5	184	1.1	2.1	195	5.5E−05
Bradi1g34670.1	BdGT61_12	1.2	2.1	187	0.9	2.1	241	1.5	2.6	183	1.5	2.0	133	1.4E−04
Bradi2g33980.1	BdBAHD04	21.4	37.7	176	21.1	40.6	193	20.0	40.3	201	26.1	37.9	145	2.5E−06
Bradi1g35736.1	cinnamoyl-CoA reductase [ortho AtCRL1]	7.3	11.5	158	9.2	12.1	132	9.3	15.3	166	9.8	18.5	188	5.0E−06
Bradi1g76460.1	GT family 77 [CAZy]	4.4	6.6	151	4.8	8.3	173	4.1	7.2	175	4.9	7.5	153	2.3E−04
Bradi2g01387.1	BdGT61_15	25.8	39.0	151	22.3	45.9	205	23.2	38.9	168	28.8	39.7	138	2.7E−05
Bradi4g27360.1	BdGT61_10	20.6	31.1	151	17.3	34.6	200	19.1	32.8	172	23.6	36.3	154	1.3E−06
Bradi3g16530.1	caffeoyl-CoA methyltransf. (CCoAMT) [PMN]	66.7	98.6	148	60.0	110.2	184	83.2	117.6	141	94.5	118.0	125	8.7E−05
Bradi1g19160.1	BdGT61_4 Clade B	13.2	19.2	145	14.7	22.1	150	15.1	20.8	138	18.6	19.2	104	4.2E−03
Bradi1g40997.1	GT family 65R [identified Nikolovski et al. 2012]	7.8	11.3	145	9.1	12.2	134	8.4	10.3	122	8.8	9.2	105	6.4E−03
Bradi2g04220.1	GT family 65R [identified Nikolovski et al. 2012]	11.0	15.8	144	10.3	15.4	149	12.6	12.8	102	12.7	13.6	107	1.3E−03
Bradi3g06480.1	cinnamyl alcohol dehydrog.(CAD) [ortho OsCAD2]	180.5	253.7	141	163.2	260.9	160	184	274.7	149	210.9	311.2	148	2.1E−04
Bradi2g43520.1	BdBAHD05 BdAT1	21.6	30.4	140	21.4	29.5	138	24.1	30.1	125	25.1	30.2	120	3.4E−04
Bradi1g34550.4	GT family 64 [CAZy]	4.8	6.7	140	4.6	6.1	132	5.5	6.7	121	5.1	8.7	170	1.4E−03
Bradi2g34240.1	cellulose synthase [ortho OsCESA1]	72.0	98.7	137	71.0	101.5	143	94.0	102.1	109	89.9	108.5	121	1.5E−05
Bradi2g61230.1	BdGT61_6 Clade B	3.1	4.2	135	3.2	5.1	162	3.1	4.9	160	4.0	6.8	171	3.7E−04
Bradi5g18377.3	GT family 65R [identified Nikolovski et al. 2012]	21.7	29.3	135	24.8	32.3	130	24.8	29.1	117	23.6	26.7	113	1.2E−03
Bradi1g76260.1	Expansins family [ortho OsEXLA1]	33.4	44.6	134	40.8	50.4	123	26.8	41.2	154	31.0	45.1	146	6.2E−03
Bradi1g53207.1	cellulose synthase [ortho OsCESA6]	12.5	16.6	133	11.6	16.5	143	12.8	15.1	118	13.7	16.6	121	2.5E−05
Bradi2g26590.1	BdGT61_14 Clade B	13.4	17.7	132	11.5	18.9	164	12.7	18.6	147	12.1	16.2	134	4.3E−07
Bradi4g04430.3	GT family 31 [CAZy]	2.8	3.7	132	2.7	4.5	166	3.4	6.7	200	4.2	7.6	184	6.9E−04
Bradi2g43510.1	BdBAHD03p1	19.7	25.1	128	17.8	27.6	155	18.1	29.3	162	21.7	24.7	114	3.1E−04
Bradi2g37970.1	xylan synthase component [ortho AtIRX9]	18.4	23.4	127	14.1	21.5	152	18.7	20.8	111	20.4	21.3	104	3.7E−04
Bradi2g55250.1	hydroxycinnamoyl-coA shikimate transf. [PMN]	3.1	3.9	127	2.6	6.4	249	3.1	6.7	215	3.2	4.3	134	2.2E−03
Bradi1g01750.1	GT family 77 [CAZy]	29.6	36.5	123	23.6	37.7	160	28.6	42.3	148	28.3	40.9	145	5.0E−05
Bradi3g39420.1	caffeoyl-CoA methyltransf. (CCoAMT) [PMN]	218.3	266.2	122	189.3	274.1	145	203	276.9	136	202.5	268.2	132	3.0E−04
Bradi3g05750.1	4-coumarate–CoA ligase (4CL) [ortho At4CL1]	20.0	24.2	121	14.3	21.8	153	17.2	24.3	141	19.4	23.0	118	3.7E−05
Bradi2g05480.1	BdBAHD01 ortho SvBAHD01	146.9	172.8	118	117.6	162.3	138	103	179.7	174	149.5	171.5	115	3.6E−03
Bradi1g64950.1	GT family 34 [CAZy]	35.5	41.0	116	29.5	39.9	135	30.7	42.0	137	34.6	38.5	111	4.1E−04
Bradi2g01480.1	BdGT61_5	61.4	70.5	115	46.7	64.1	137	52.9	64.3	122	56.1	60.2	107	1.7E−03
Bradi2g43890.5	GT family 4R [identified Nikolovski et al. 2012]	8.9	10.0	113	7.7	9.8	127	8.4	8.9	105	8.4	9.9	117	9.5E−03
Bradi1g64830.3	GT8 family [ortho AtGATL7]	15.3	16.5	108	11.5	18.0	157	11.5	20.6	179	12.9	15.5	120	2.2E−04
Bradi5g24290.1	xylan synthase component [ortho AtIRX14-L]	31.2	33.6	108	25.1	35.2	140	31.7	40.8	128	35.7	40.8	114	7.0E−03

Overall significance of MeJA treatment *P* values are corrected for false-discovery rate by Benjamini–Hochberg method. Genes are ordered by descending fold change at 24-h

Different members of the GT77 and GT31 families are among the most down-regulated cell-wall transcripts, along with those from GT37 (Table 2). These three GT families all contain members that glycosylate cell-wall proteins, as well as some involved in pectin decoration. Extensins and an expansin are moderately down-regulated, as are some CSLA, CSLE, and CSLH family transcripts (Table 2).

**Table 2:** Transcript abundance in FPKM (average of 4 reps) of down-regulated cell-wall genes after 1-, 2-, 4-, or 8-day treatment with 50-µM MeJA (JA) compared to a mock control (MC)

Transcript	Candidate name/putative function [source]	24 h			48 h			4 days			8 days			Overall JA *P* value
		MC	JA	JA/MC (%)	MC	JA	JA/MC (%)	MC	JA	JA/MC (%)	MC	JA	JA/MC (%)	
Bradi3g14370.1	GT31 family [ortho AtB3GALT20]	3.6	3.1	87	3.6	3.3	92	4.0	3.0	75	4.0	3.0	76	6.1E−03
Bradi2g33090.1	GT31 family [ortho AtB3GALT12]	7.0	6.0	86	7.3	5.6	77	7.7	4.9	64	6.3	5.0	80	2.2E−03
Bradi3g44420.1	xylan synthase component [ortho GT47 AtIRX10L-like]	36.1	31.1	86	42.4	29.0	68	48.6	31.2	64	40.8	34.3	84	5.3E−04
Bradi3g25658.1	GT family 2 CSLA [CAZy]	7.1	5.9	83	8.4	6.5	77	9.6	7.3	76	8.8	6.7	77	4.1E−03
Bradi3g14860.1	GT family 31 [CAZy]	19.7	15.8	80	17.9	16.2	90	18.8	15.1	80	19.6	16.5	84	7.9E−04
Bradi1g54620.1	GT31 family [ortho AtB3GALT19]	3.3	2.5	77	3.3	2.4	72	2.8	2.2	78	3.4	1.7	51	1.8E−03
Bradi1g75450.1	GT47 family [ortho AtKAM1]	3.6	2.6	74	3.4	2.5	74	4.9	3.0	61	3.9	2.6	67	1.9E−03
Bradi3g47480.1	GT family 47 [CAZy]	5.3	3.9	73	4.6	3.3	73	4.6	2.7	60	4.6	2.5	55	5.4E−03
Bradi2g58994.2	GT family 77 [CAZy]	0.8	0.6	71	1.2	0.6	49	2.8	0.3	12	2.6	0.9	34	7.9E−06
Bradi4g33090.1	GT family 2 CSLE [CAZy]	8.3	5.7	69	7.9	6.7	85	8.9	5.4	61	5.8	5.5	95	9.7E−03
Bradi1g07900.1	Extensins family [Panther]	7.0	4.4	62	5.2	4.3	83	10.4	4.6	45	10.5	3.6	35	7.5E−04
Bradi1g35830.1	Expansins family [ortho OsEXPA16]	6.0	3.7	62	4.3	3.6	83	2.6	1.8	71	3.4	1.8	55	5.9E−03
Bradi5g18927.1	GT47 family [ortho AtXLT2]	1.4	0.8	60	1.5	0.7	47	1.2	0.8	69	1.0	0.6	54	1.6E−03
Bradi5g10130.1	β-(1,3;1,4)-glucan synthase [ortho OsCslH1]	33.5	20.0	60	33.6	24.8	74	23.2	16.6	71	21.4	16.4	77	8.8E−03
Bradi1g64560.1	GT family 34 [CAZy]	2.6	1.5	59	1.6	1.0	61	1.6	0.9	60	1.4	0.7	51	5.9E−03
Bradi5g21550.1	Cinnamyl alcohol dehydrogenase (CAD) [PMN]	5.1	2.9	56	4.1	2.0	49	3.8	2.5	68	4.4	2.1	48	5.8E−04
Bradi2g48710.1	GT8 family [ortho AtGAUT15]	1.0	0.6	55	0.8	0.3	38	1.0	0.6	56	1.2	0.7	57	2.0E−03
Bradi1g29515.1	Extensins family [Panther]	6.8	3.2	47	4.4	2.7	61	7.4	3.6	48	6.4	2.4	38	3.7E−04
Bradi1g46037.1	GT family 37 [CAZy]	2.4	1.0	42	1.7	1.1	67	2.2	0.7	30	1.2	0.8	64	1.6E−03
Bradi1g46030.1	GT family 37 [CAZy]	0.7	0.2	34	0.6	0.3	59	1.2	0.3	24	1.0	0.0	4	1.3E−03
Bradi4g30955.2	GT family 31 [CAZy]	1.9	0.6	32	1.4	0.5	37	2.1	0.9	42	1.4	0.8	56	4.2E−03
Bradi2g59017.1	GT family 77 [CAZy]	0.5	0.1	17	0.4	0.0	9	1.1	0.2	17	1.1	0.2	15	1.1E−03
Bradi4g32160.1	GT family 37 [CAZy]	1.0	0.1	11	0.9	0.1	8	0.9	0.3	29	0.6	0.1	23	6.4E−05

Overall significance of MeJA treatment *P* values are corrected for false-discovery rate by Benjamini–Hochberg method. Genes are ordered by descending fold change magnitude at 24 h

**Fig. 6:**
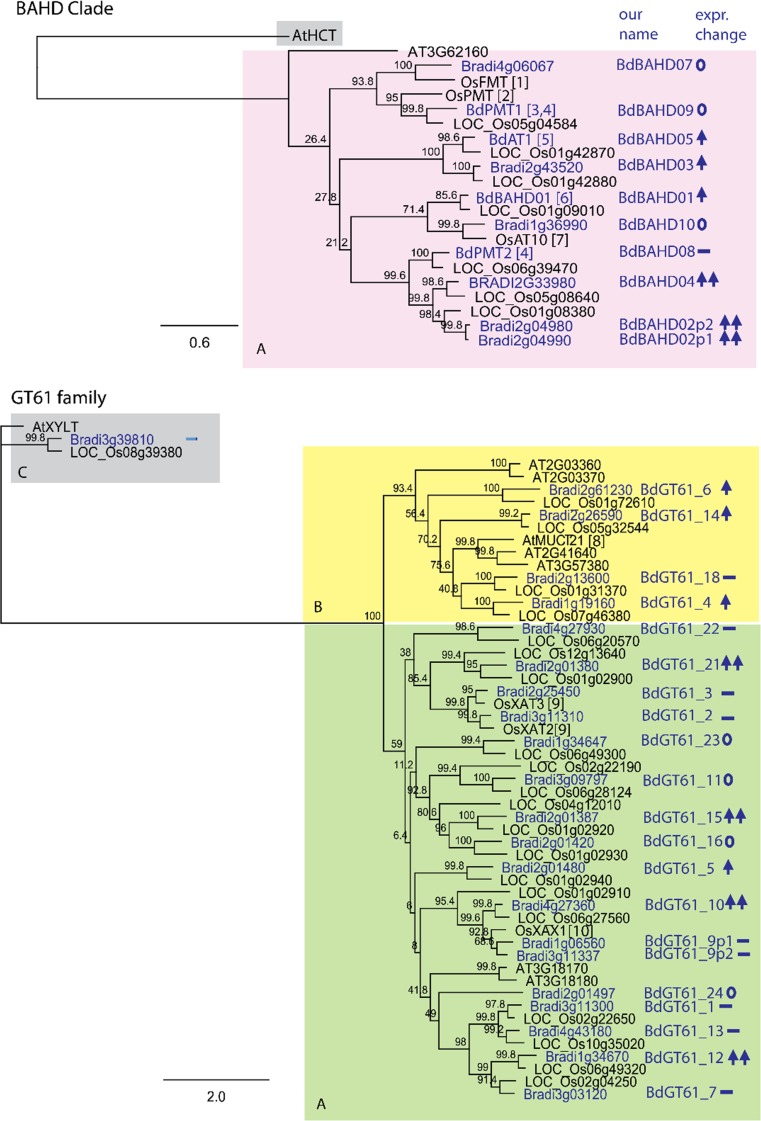
Phylogenetic trees of BAHD Clade and GT61 family genes indicating effect of MeJA treatment on Brachypodium genes in callus: ↑↑ up-regulation by > twofold, ↑ up-regulation by < twofold,—no significant change, 0 not expressed (< 1 FPKM). Outgroups used to root trees are in grey blocks. Support for topology is shown as percentage of 500 bootstrap runs. Only sub-clade A (pink block) out of BAHD Clade genes (as defined Molinari et al. (2013)) is shown as sub-clade B genes were not expressed. GT61 family clades A (yellow) B (green) C (grey) are shown (as defined Anders et al. (2012)). Named genes on tree have evidence on their role from [1] Karlen et al. (2016) [2] Withers et al. (2012) [3] Petrik et al. (2014) [4] Sibout et al. (2016) [5] Buanafina et al. (2016) [6] de Souza et al. (2018) [7] Bartley et al. (2013) [8] Voiniciuc et al. (2015) [9] Anders et al. (2012) [10] Chiniquy et al. (2012)

Several candidate genes for *p*CA and FA esterification to AX in the BAHD Clade and GT61 families increased significantly in response to MeJA (Table 1), whilst others did not respond or were not expressed. Due to the high level of replication and consistency of response to MeJA across timepoints, we were able to detect modest up-regulation (1.3–1.9-fold) with a high level of statistical certainty and distinguish this from more substantial up-regulation (≥ 2.0-fold) (Table 1). We summarise these responses to MeJA for all the BAHD candidate Clade genes and GT family genes in Fig. 6, along with their phylogenetic relationship to genes that have evidence on their role.

Our focus here was on cell-wall changes, particularly in HCA composition induced by MeJA, rather than to study JA signalling in Brachypodium callus. However, the detailed, strand-specific RNA-seq transcriptome set that we generated can also be mined for this purpose. As well as details of all DEGs (Table S4), we have made all raw data available at ArrayExpress accession E-MTAB-5413.

## Discussion

### Response of Brachypodium callus cell wall to MeJA

JA signalling induces a slowing of growth and a priming of defence responses. The cell-wall component of these responses includes a large increase in transcripts and enzyme activities for lignin biosynthesis and for generation of ROS which can induce cross-linking of cell-wall components. In primary cell walls, increased cross-linking can stop cell expansion, and it can strengthen all cell walls against attack. The Brachypodium callus used for this study grew rapidly (Fig. S1) and the transcriptome analysis showed low expression of secondary cell-wall-specific cellulose synthase CESA genes (*Bradi2g49912*, *Bradi3g28350*, and *Bradi4g30540* in Table S5) indicating that very little or no secondary cell walls were present, although it did contain lignin which is consistent with the previous findings (Rancour et al. 2012). MeJA treatment caused a marked slowing of growth and some changes in cell-wall composition. We found some changes in lignin amount, but these were small and inconsistent (Figs. 4b; 5f); JA treatment does not always induce lignin increases in plants (Napoleao et al. 2017), although monolignol synthesis was increased in cell cultures (Pauwels et al. 2008). No significant changes in polysaccharide composition of the cell walls during the 8-day time course were induced by 50-µM MeJA except for a decrease in hemicellulosic glucose (Fig. 4); however, 17 days after 50-µM MeJA treatment, we also observed significant increases in xylose, arabinose, and cellulosic glucose (Fig. 2).

We had hypothesised that AX-*p*CA and AX-FA would be up-regulated in response to MeJA based on transcript responses to JA in rice (Sato et al. 2013). Wall-bound *p*CA increased strongly in MeJA-treated callus, increasing five–tenfold after 17 days of treatment with 50-µM MeJA (Fig. 1). In our two time-course experiments, wall-bound *p*CA was significantly greater than the control samples after 24-h MeJA treatment, and was 70–110% increased after 8-day treatment (Figs. 3a,5a); as MeJA did not affect the amount of arabinose or xylose in the callus cell walls during this period, bound *p*CA per unit AX was similarly increased. A smaller increase was observed in FA monomer which was significant at 17 days but not during the 8-day time-course experiments. FA dimers were increased by MeJA in all experiments (Figs. 1, 3, 5) and this effect was seen in all individual dimers measured (Table S2). Our direct measurement of Ara*f*-FA and Ara*f*-*p*CA (Fig. 5d, e) was consistent with the assumption that all or nearly-all ester-linked cell-wall FA and *p*CA were derived from AX-FA and AX-*p*CA in the callus tissue. Therefore, MeJA induces large increases in AX-*p*CA whilst having only a small effect on AX-FA monomer in Brachypodium callus.

### Cell-wall transcript responses to MeJA

Overall, we observed effects on cell-wall transcripts consistent with changes in cell-wall composition induced by MeJA. Modest increases in amounts of cellulose and AX (Fig. 2) were preceded by increases in CESA and GT43 transcript abundance (Table 1) and a decrease in hemicellulosic glucose (Figs. 2, 4) was accompanied by a decrease in *Bradi5g10130* CSLH2 transcript abundance (Table 2). CSLH genes encode (1,3;1,4)- β-glucan synthases (Doblin et al. 2009); the most abundantly expressed (1,3;1,4)- β-glucan synthase in most barley tissues is CSLF6 (Burton et al. 2008), but in Brachypodium callus, this was similarly expressed to CSLH2 and unaffected by MeJA.

Our original motivation for studying the response to JA in Brachypodium was the large up-regulation of certain BAHD (*OsBAHD02*, *OsBAHD04*) and GT61 genes (*OsGT61_21*) in rice seedlings following JA treatment (Sato et al. 2013); we found the same effects here for the Brachypodium orthologues of these genes (Fig. S4), showing that the responses are common to both systems. We also found three further up-regulated GT61 genes in Brachypodium (*BdGT61_12*, *BdG61_15*, *BdGT61_10*), but only one orthologue (*OsGT61_10*) of these was up-regulated in rice (Fig. S4). The greatest relative up-regulation shown of these, and of all cell-wall genes except for one CCR gene, were the two paralogs *BdBAHD02p1*, *p2* (*Bradi2g04980*, *Bradi2g04990*; most likely the result of a recent tandem duplication) (Table 1). Interestingly, the orthologue of these genes in switchgrass *Pavir.Eb00373* is substantially down-regulated upon induction of secondary cell-wall associated lignification (Rao et al. 2017), perhaps suggesting that its role is restricted to primary cell walls. One other closely related BAHD, *BdBAHD04* (*Bradi2g33980*) was more highly expressed and was also up-regulated by MeJA. Three further BAHDs (*BdBAHD01*, *03*, *05*) in the clade were significantly up-regulated but by less than twofold (Table 1). These results and their relationship to other BAHDs with evidence of function are summarised in Fig. 6. The ortholog of the *OsAT10* gene previously implicated to be responsible for the addition of *p*CA to AX was not expressed in callus. Genes responsible for *p*-coumarylation of monolignols *BdPMT1* and *BdPMT2* had, respectively, zero and low expression and were not up-regulated by MeJA. The orthologue of *OsFMT* that is putatively responsible for feruloylation of monolignol was not expressed. Genes with the strongest evidence for a role in AX feruloylation *BdBAHD01* and *BdAT1* (our *BdBAHD05*) were moderately up-regulated. Therefore, it seems likely that at least one of the most up-regulated genes *BdBAHD02p1*, *BdBAHD02p2,* and/or *BdBAHD04* performs the same molecular function as *OsAT10*, and their up-regulation accounts for the large rise in AX-*p*CA observed in response to MeJA. These genes have most sequence similarity to *BdPMT2* (Fig. 6) which may suggest that relatively few amino acid residue changes in these enzymes are required to alter the acceptor specificity between Ara*f* and monolignol.

Some GT61 Clade A genes (*XAT1, 2* and *3*) encode arabinosyl transferases (Anders et al. 2012). As outlined above, we favour a model, where some GT61 proteins are responsible for HCA–arabinosyl transfer onto xylan, explaining the decreases in bound *p*CA and FA seen in the *xax1* mutant (Chiniquy et al. 2012). Neither of the two Brachypodium orthologs of *XAX1 Bradi1g06560* nor *Bradi3g11337* showed significant change in gene expression when treated with MeJA; however, the closely related *Bradi4g27360* was substantially up-regulated (Table 1; Fig. 6). Three other Clade A GT61 genes were up-regulated by twofold or more: *Braidi2g01380*, *Bradi2g01387*, and *Bradi1g34670* (Table 1; Fig. 6) and could, therefore, be considered candidates for an HCA-Ara addition step. Since both FA and *p*CA were decreased in the *xax1* mutant (Chiniquy et al. 2012), it may be that GT61-encoded enzymes are not specific for either FA-Ara or *p*CA-Ara. Other GT61 genes in Clades A and B that were less up-regulated (< 2.0-fold) show similar up-regulation to GT43 genes encoding IRX9 and IRX14 homologues (Table 1) that are involved in xylan backbone synthesis, so may be part of a general increase in AX synthesis (Fig. 2).

**Fig. 7:**
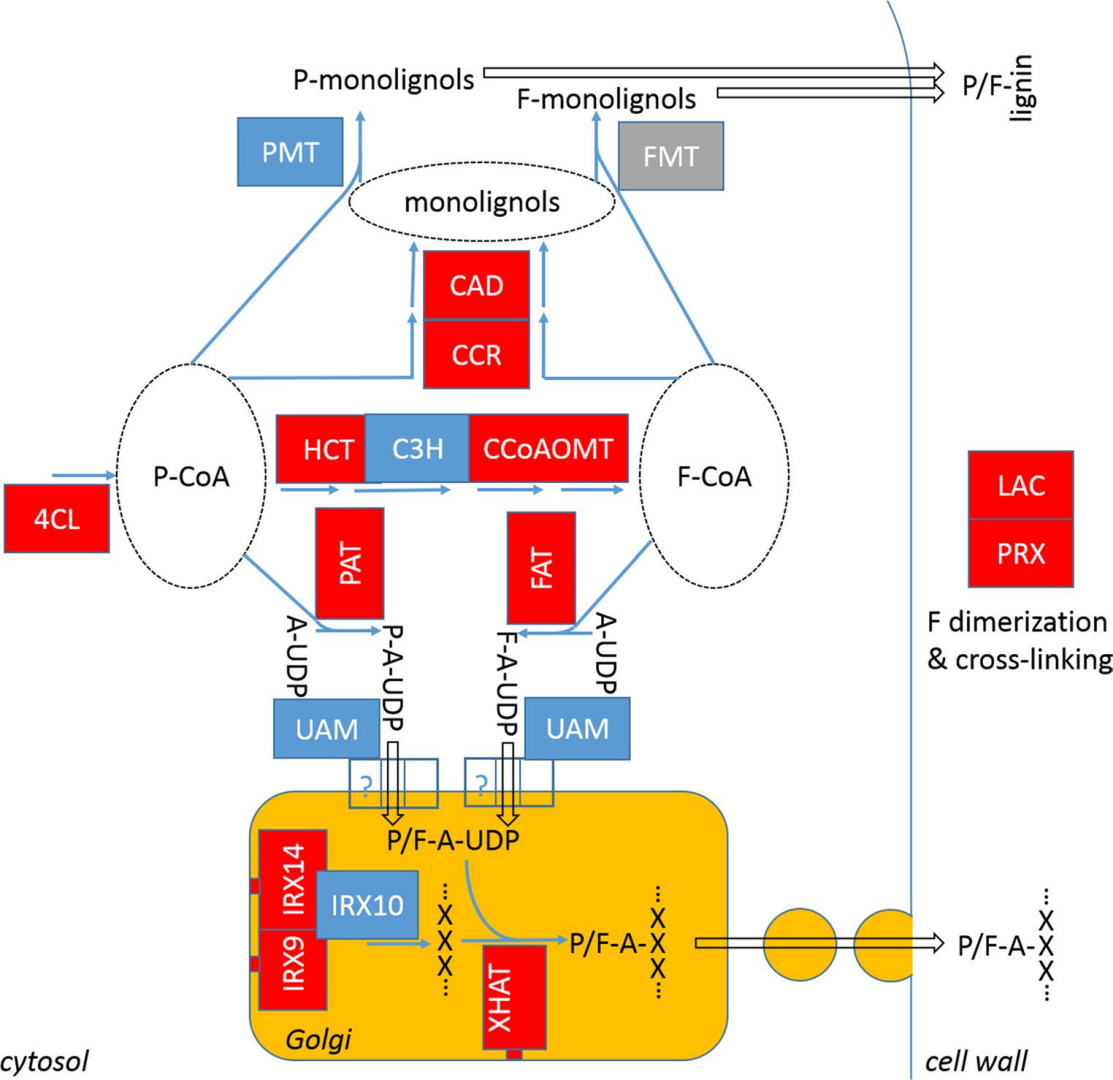
Possible pathways to cell wall for ester-linked HCA (pCA and FA represented as *P* and *F,* respectively; *P*/*F* denotes *P* or *F*). X denotes xylosyl and A arabinofuranosyl residues of AX. Enzymes are shown as rectangles coloured according to response of putative encoding transcripts: grey, not expressed; blue, no significant response to MeJA, red, up-regulated by MeJA. Protein marked is unknown UDP-arabinofuranose transporter. In this model, *BdBAHD01*, *BdBAHD05* encode feruloyl arabinosyl transferases (FATs) and *BdBAHD02p1*, *BdBAHD02p1*, *BdBAHD03* and *BdBAHD04* could all encode p-coumaroyl arabinosyl transferases (PATs) or FATs, and any of GT61 Clade A genes shown in Fig. 6 could encode xylan hydroxycinnamoyl arabinosyl transferase (XHAT)

Genes in the phenylpropanoid pathway are some of the most up-regulated genes in our cell-wall set, although lignin was only moderately increased (Figs. 4, 5). The early steps in the pathway also generate *p*CA-CoA and FA-CoA precursors for ester-linked HCAs in the cell wall and other phenolics, but the amounts of these are small compared to lignin. This increase in transcripts may, therefore, be more part of defence priming allowing rapid lignification in response to additional cues. Along with RNAi studies suppressing BAHD and GT61 genes resulting in decreased cell-wall HCAs discussed above, RNAi suppression of UAM genes has also been shown to have this effect (Rancour et al. 2015); UAM proteins interconvert UDP-Arap to UDP-Araf and are located on the outside of the Golgi (Rautengarten et al. 2011). UAM transcripts are abundant but not up-regulated by MeJA in our system. Possible pathways for cell-wall ester-linked HCAs are shown in Fig. 7, highlighting that the enzymes for which putatively encoding transcripts are up-regulated in Brachypodium callus. In this model, genes in the BAHD candidate Clade A (Fig. 6) encode the four enzymes which together account for all ester-linked HCA in grass cell walls by the addition of *p*CA or FA to monolignol (PMT, FMT) or to Araf (PAT, FAT). In addition to cell-wall enzymes, class III peroxidase and laccase enzymes that generate ROS responsible for oxidative coupling in the cell wall are also depicted. Some transcripts for these are massively up-regulated in response to MeJA (Table S4); the increased FA dimerization that we observed suggests increased oxidative coupling did occur in the cell walls (Figs. 1, 3, 5). It has previously been suggested that *p*CA in grass cell walls (mostly on lignin) acts as an “oxidation catalyst” for S-lignin polymerisation by radical transfer (Ralph 2010); perhaps, AX-*p*CA could play a role in facilitating or accelerating AX-FA dimerization. This would seem to fit with a general picture of the effect of JA signalling on cell walls, where the largest responses are often increased cross-linking as part of decreased cell expansion and enhanced defence.

## Conclusion

Our results show that increased AX-*p*CA is the largest relative response to MeJA in cell-wall components measured in Brachypodium callus. The accompanying large up-regulation of candidate BAHD and GT61 genes is consistent with a role for these in the synthesis of this linkage. Since the callus system is amenable to transformation, we plan to investigate this by direct manipulation of these genes’ expression.

### *Author contribution statement*

LSH, TKP, JF, SJM and RACM conceived and designed research. LSH, TKP and JF conducted experiments; LVM and RS contributed mass spectrometry and lignin analyses, respectively. LSH, TKP, JF, LVM and RACM analysed data. LSH and RACM wrote the manuscript. All authors read and approved the manuscript.

## Electronic supplementary material

Below is the link to the electronic supplementary material.

Supplementary material 1 (DOCX 2660 kb)

Supplementary material 2 (XLSX 3183 kb)

Supplementary material 3 (XLSX 227 kb)

## Abbreviations

AIR: Alcohol-insoluble residue
Araf: Arabinofuranose
AX: Arabinoxylan
BAHD: Superfamily of acyl-coA transferases
DEG: Differentially expressed gene
diFA: Ferulic acid dimer
FA: Ferulic acid
GT: Glycosyltransferase
HCA: Hydroxycinnamic acid
JA: Jasmonic acid
MeJA: Methyl jasmonate
*p*CA: *para*-coumaric acid
ROS: Reactive oxygen species
