# Supplementary material for: Response of cell-wall composition and RNA-seq transcriptome to methyl-jasmonate in Brachypodium distachyon callus
Source: Planta. 2018 Aug 9;248(5):1213–29. doi: 10.1007/s00425-018-2968-9 (PMC6182315; doi:10.1007/s00425-018-2968-9)
Supplement: Supplementary file 1 — Supplementary material 1 (DOCX 2660 kb) [file 425_2018_2968_MOESM1_ESM.docx]

**
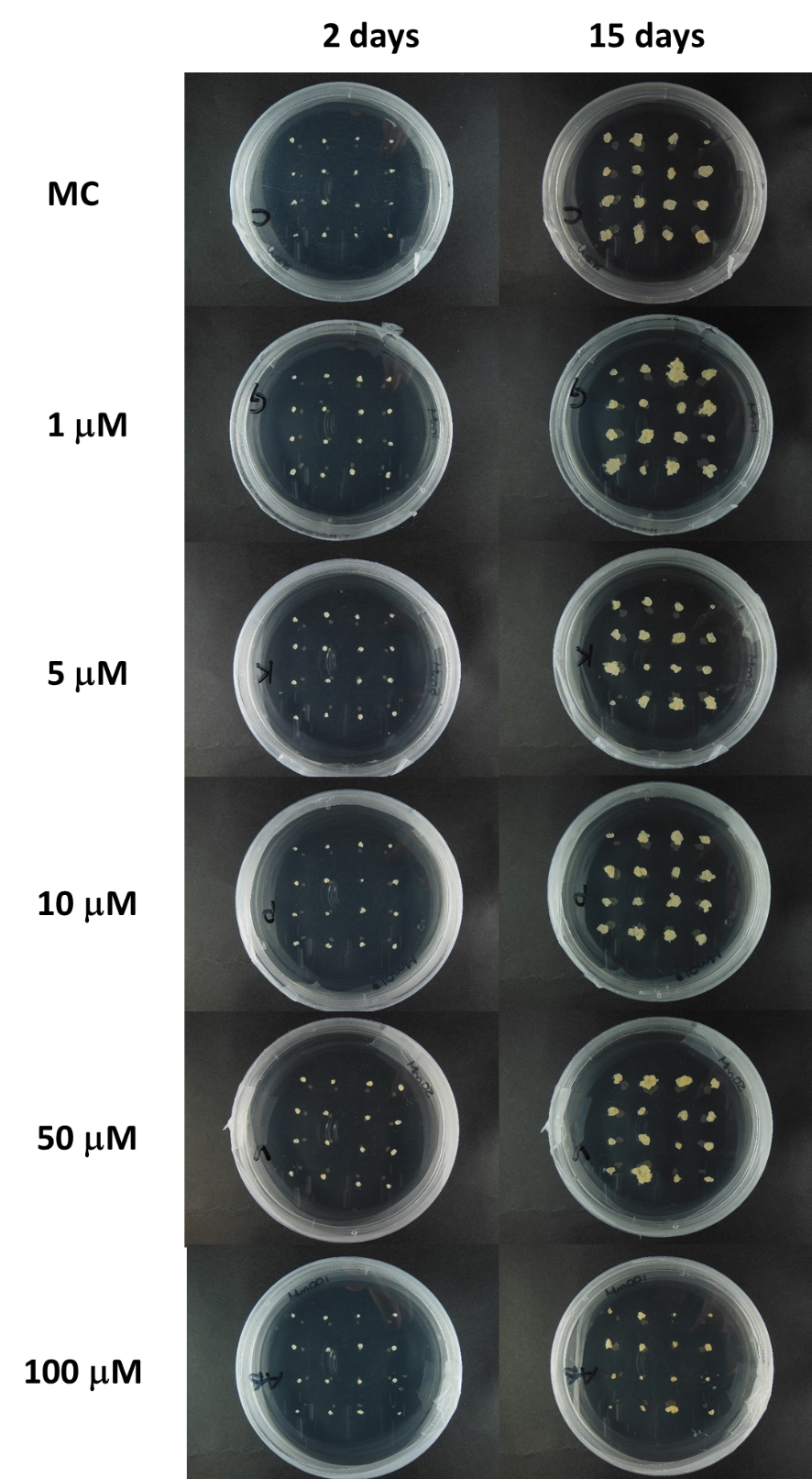
**
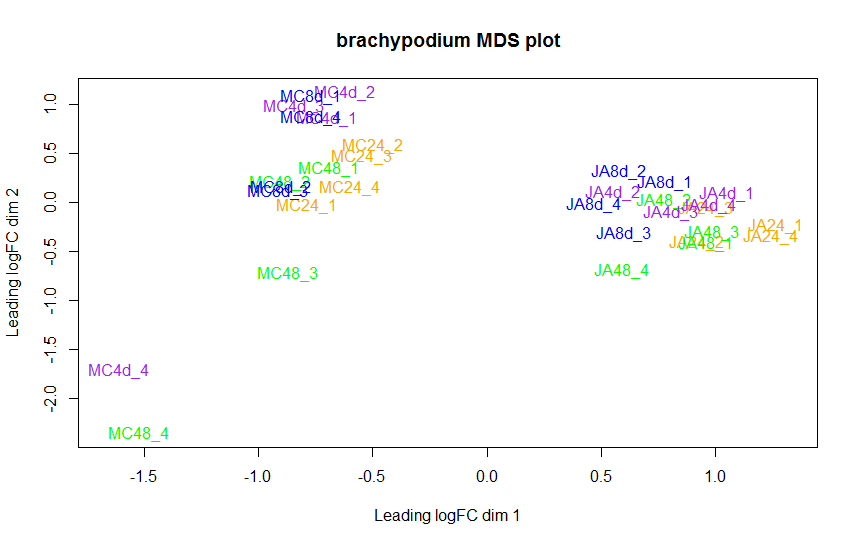


**Figure S1.** Plates of Brachypodium callus from Expt. 1 at 2 and 15 days after transfer to medium with different concentrations of MeJA. Slower growth of callus is evident at high MeJA concentrations.

**Figure S2.** Multidimensional scaling factor plot showing clustering of RNA sequencing samples of Brachypodium treated with 50 µM MeJA (JA) for 24 h, 48 h, 4 d or 8 d, compared to a mock control. Four biological replicates were analysed (1-4). Plot was generated in R Studio by calculating leading log fold change (FC) from effective counts of reads, for genes with counts per million > 1 in 3 or more samples


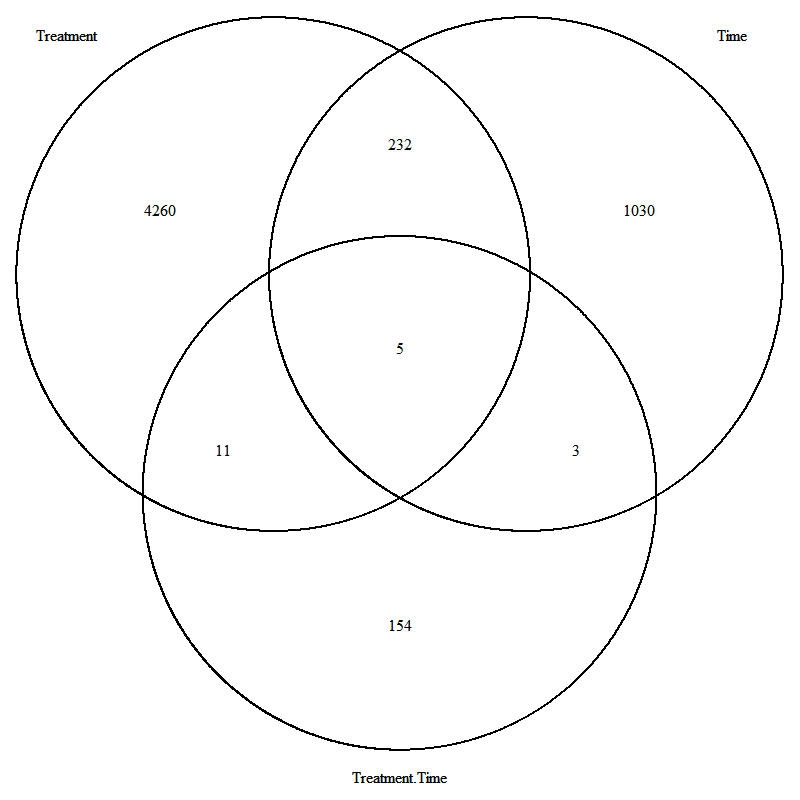


**Figure S3.** Venn diagram showing counts of differentially expressed genes (DEGs) for ANOVA factors treatment (MeJA), time (1-8 d) and the treatment.time interaction. Samples were treated with 50 µM MeJA or a mock control for 1, 2, 4 or 8 d

**
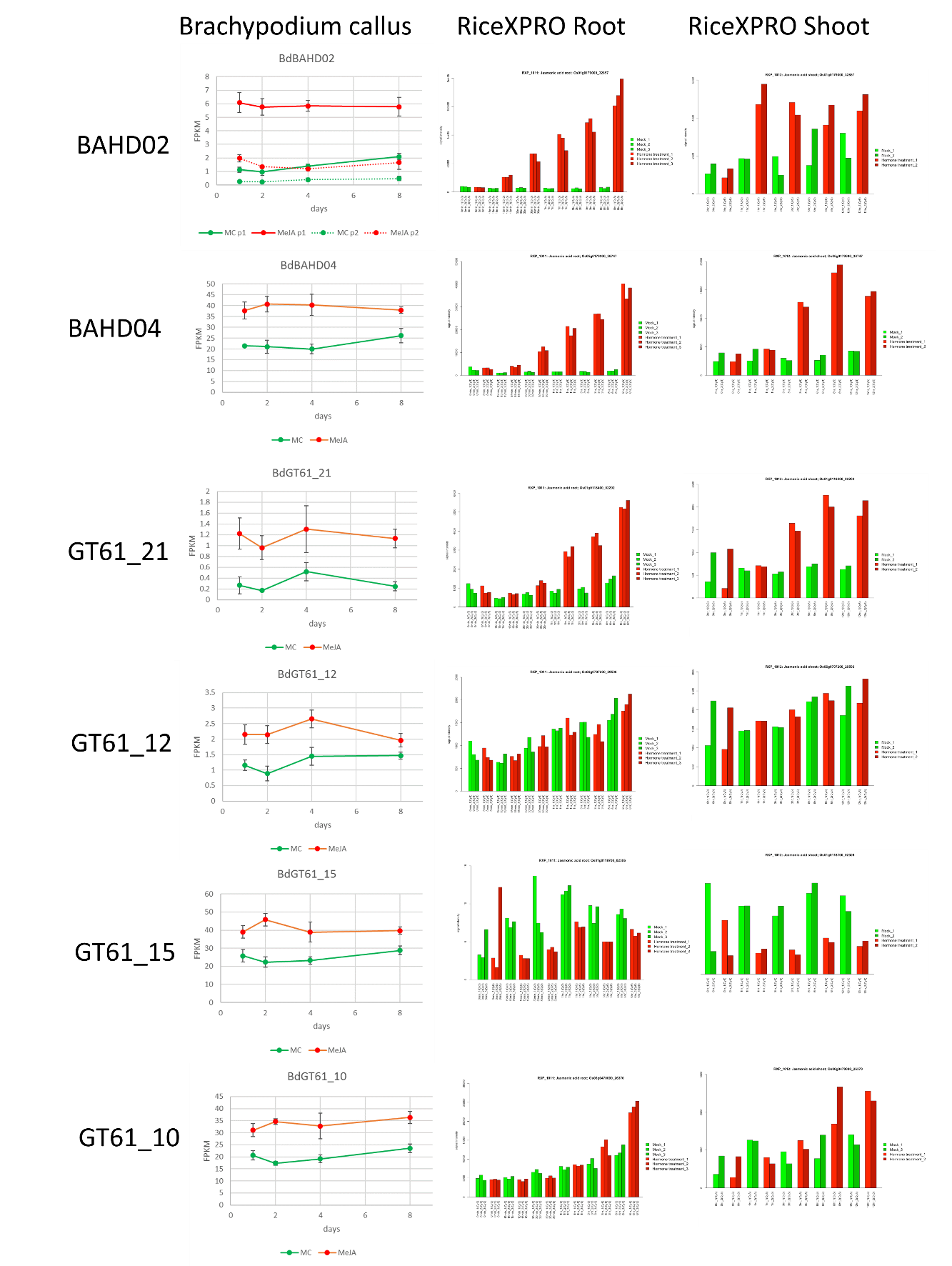
Figure S4.** Comparison of expression of up-regulated BAHD and GT61 genes determined here in Brachypodium callus with response of orthologues in rice seedlings to JA from RiceXPro expression database (Sato et al. 2013).

**Table S1** The average proportion of the dry weight (± SE, *n*=4) of alcohol insoluble residue (AIR), and destarched AIR, in control and 50 µM MeJA treated Brachypodium callus samples. Data is from samples from experiment 3.

|  | AIR (% of DW) | | Destarched AIR (% of DW) | |
| --- | --- | --- | --- | --- |
|  | control | MeJA | control | MeJA |
| 24 h | 39.5 (± 0.8) | 40.9 (± 0.6) | 18.6 (± 0.8) | 18.5 (± 3) |
| 48 h | 40.9 (± 0.5) | 42.2 (± 0.7) | 22.2 (± 0.7) | 22.3 (± 0.6) |
| 4 d | 40.8 (± 0.7) | 41.5 (± 0.5) | 20.5 (± 0.8) | 21.3 (± 0.5) |
| 8 d | 40.3 (± 1.1) | 41.4 (± 0.8) | 20.6 (± 1.1) | 21.5 (± 0.8) |

**Table S2** Individual FA dimer content from all experiments. *, ** effect sig. at *P*<0.05, 0.01. Amounts of MeJA-treated samples are expressed as percentage of control amounts. AT = aryltetralin; BF = benzofuran.

|  | diF8-8AT (a) | diF8-8 | diF8-5 | | diF5-5 | | diF8-0-4 | | diF8-5BF | | | diF8-O-4 & diF8-5BF (b) |  |
| --- | --- | --- | --- | --- | --- | --- | --- | --- | --- | --- | --- | --- | --- |
| Experiment 1 (µg mg-1 DW, 17 days MeJA) | | | | | | | | | | | | |  |
| control |  | 0.13 | 0.19 | | 0.21 | |  | |  | | | 0.39 |  |
| 1 µM MeJA |  | 128% * | | 130% * | | 126% | |  | |  | 130% * | | |
| 5 µM MeJA |  | 123% * | | 119% | | 112% | |  | |  | 131% * | | |
| 10 µM MeJA |  | 130% * | | 127% * | | 120% | |  | |  | 128% * | | |
| 50 µM MeJA |  | 140% ** | | 160% ** | | 158% ** | |  | |  | 169% ** | | |
| 100 µM MeJA |  | 133% ** | | 158% ** | | 198% ** | |  | |  | 187% ** | | |
| Experiment 2 (µg mg-1 DW, 17 days MeJA) | | | | | | | | | | | | |  |
| control |  | 0.07 | 0.16 | | 0.20 | | 0.15 | | 0.16 | | |  |  |
| 1 µM MeJA |  | 133% | 137% | | 126% | | 129% | | 136% | | |  |  |
| 5 µM MeJA |  | 138% | 148% | | 140% | | 150% | | 127% | | |  |  |
| 10 µM MeJA |  | 143% | 157% | | 163% | | 160% | | 145% | | |  |  |
| 50 µM MeJA |  | 267% ** | 300% ** | | 351% ** | | 354% ** | | 309% ** | | |  |  |
| 100 µM MeJA |  | 265% ** | 304% ** | | 420% ** | | 397% ** | | 317% ** | | |  |  |
| Experiment 3 (µg mg-1 AIR, 50 µM MeJA) | | | | | | | | | | | | |  |
| 1d control |  | 0.45 | 0.52 | | 0.27 | | 0.59 | | 0.93 | | |  |  |
| 2d control |  | 0.41 | 0.47 | | 0.44 | | 0.66 | | 0.64 | | |  |  |
| 4d control |  | 0.56 | 0.66 | | 0.46 | | 0.75 | | 0.78 | | |  |  |
| 8d control |  | 0.43 | 0.53 | | 0.37 | | 0.67 | | 0.75 | | |  |  |
| 1d MeJA |  | 92% | 96% | | 119% | | 111% | | 100% | | |  |  |
| 2d MeJA |  | 127% | 130% | | 109% | | 111% | | 112% | | |  |  |
| 4d MeJA |  | 95% | 97% | | 97% | | 97% | | 110% | | |  |  |
| 8d MeJA |  | 94% | 103% | | 145% | | 121% | | 109% | | |  |  |
| Experiment 4 (µg mg-1 AIR, 50 µM MeJA) | | | | | | | | | | | | |  |
| 1d control | 0.14 | 0.43 | 0.64 | | 1.18 | |  | |  | | | 1.47 |  |
| 2d control | 0.15 | 0.45 | 0.65 | | 1.28 | |  | |  | | | 1.55 |  |
| 4d control | 0.14 | 0.38 | 0.57 | | 1.12 | |  | |  | | | 1.33 |  |
| 8d control | 0.15 | 0.44 | 0.61 | | 1.32 | |  | |  | | | 1.49 |  |
| 1d MeJA | 98% | 98% | 103% | | 98% | |  | |  | | | 103% |  |
| 2d MeJA | 97% | 99% | 103% | | 100% | |  | |  | | | 105% |  |
| 4d MeJA | 107% * | 113% * | 117% * | | 120% * | |  | |  | | | 119% * |  |
| 8d MeJA | 116% * | 124% | 135% * | | 126% | |  | |  | | | 131% * |  |

(a) only measured in Expt 4. (b) peaks not resolved in Expts. 1&4.

**Table S3 Ester-linked HCA content of supernatant and pellet following mild acidolysis.** The mean percentage of hydroxycinnamic acids associated with the supernatant or pellet fraction of Brachypodium callus destarched AIR (alcohol insoluble residue) which was subjected to mild transfluoroacetic acid (TFA) (0.05 M) hydrolysis after 7 days treatment with methyl-jasmonate (MeJA).

|  | Supernatant | | Pellet | |
| --- | --- | --- | --- | --- |
|  | Control | MeJA | Control | MeJA |
| *p*CA | 93.0 | 93.0 | 7.0 | 7.0 |
| tFA monomer | 89.3 | 90.9 | 10.7 | 9.1 |
| tFA dimer | 88.6 | 93.0 | 11.4 | 7.0 |
